# The impact of phenological shifts on carbon uptake across major terrestrial biomes

**DOI:** 10.1186/s13021-026-00450-4

**Published:** 2026-05-06

**Authors:** Getachew Mehabie Mulualem, Jadunandan Dash

**Affiliations:** School of Geography and Environmental Science, University of Southampton, Southampton, SO171BJ UK

**Keywords:** Length of growing season, Gross primary productivity, Phenology, Discrete fourier transforms, Flux towers data

## Abstract

Changes in climate are altering plant growth patterns and associated phenological events like the Start of Season (SOS), End of Season (EOS), and Length of the Growing Season (LGS). However, there is limited research quantifying the impact of these changes on key vegetation-atmospheric interaction processes such as the carbon and water cycles. This study uses 914 site years of data across 132 flux tower sites in the FLUXNET2015 dataset to explore the relationships between carbon sequestration, expressed by Gross Primary Productivity (GPP), and multiple phenological variables, including LGS, changes in SOS (ΔSOS), and changes in EOS (ΔEOS). LGS explains 23% of the variability in GPP across all sites. Significant correlations were found in deciduous broadleaf forests (R² = 0.5) and evergreen needleleaf forests (R² = 0.44), while ecosystems such as shrublands, savannas, and wetlands displayed weaker connections. Changes in the SOS also affected GPP, with an earlier SOS increasing the total annual GPP. Deciduous Broadleaf Forests (R² = 0.54), Evergreen Needleleaf Forests (R² = 0.5), Grasslands (R² = 0.47) showed a significant negative association between ΔSOS and ΔGPP, whereas Croplands showed weaker correlations. Conversely, EOS variations had little impact on GPP. Upscaled to global vegetated land area these relationships suggest that each additional day in the growing season could increase carbon uptake by 1.035 Gt C yr^− 1^, while an earlier SOS by 0.93 Gt C yr^− 1^ and a one-day delay in EOS by approximately 0.65 Gt C yr^− 1^. These findings underscore the need to account for seasonal shifts and phenological changes in global carbon models.

**Supplementary Information:**

The online version contains supplementary material available at 10.1186/s13021-026-00450-4.

## Introduction

 Phenology refers to the seasonal biological events in plant life cycles, affects processes like photosynthesis and respiration [12]. Examining the connection between seasonal biological cycles and ecosystem carbon fluxes is essential for comprehending carbon cycling, especially in the context of climate change. These processes regulate the absorption of CO2 in land ecosystems. Gross Primary Productivity (GPP), a key measure of the carbon plants capture during photosynthesis, depends on these natural cycles [43]. As global temperatures increase and rainfall patterns change, the phenological cycle is impacted and the timing of significant phenological events [14, 17, 50]. In northern high latitudes, the Start of Season (SOS) is advancing several days while the End of Season (EOS) is delaying resulting in an extended growing season. Recent studies have shown this trend, with Jeganathan et al., [21] and later works by Zhang et al., [56] and Chen et al., [9] confirming the advancement of SOS in the Northern Hemisphere. Wei et al., [51] also found that due to shifts in phenological dates the relationship between plant and animal life cycles were disturbed, which influenced bird migration and other ecological processes. For instance, early spring cause plants to flower earlier, but birds might not migrate earlier, leading to a mismatch between food availability and migration timing.

Changes in phenological dates are having an increasing influence on carbon storage throughout ecosystems globally. In temperate forests, warmer temperatures are causing leaves to appear earlier in the spring, which boosts photosynthesis and may increase the ability to capture carbon [32]. In the Arctic region longer growing seasons allow plants to store more carbon within their biomass, leading to increased amounts of storage of carbon [49]. These changes are fundamentally shifting how ecosystems manage and store carbon. While phenological shifts often enhance GPP, they can also increase ecosystem respiration (RE), partially offsetting GPP gains (Piao et al., 2008; [41]). Longer growing seasons may elevate soil and plant respiration, affecting net ecosystem exchange (NEE) [2, 25]. However, generally GPP increases more than RE, resulting in a net carbon uptake [25].

Many studies have reported a relationship between phenological events and GPP [19, 22, 34]. Richardson et al., [40] has identified that a significant correlation exists between the timing of leaf-out and GPP in temperate forests. Earlier leaf-out increases carbon uptake by prolonging the photosynthetic period. Piao et al., [36] observed that the extension of growing seasons in boreal and temperate ecosystems has led to substantial increases in GPP because of rising temperatures. Kindomihou [26], indicated that in grasslands, the timing of flowering in plants influences the quantity of carbon sequestered in the soil via root turnover and decomposition. Plants that exhibited earlier flowering allocated more resources to root growth, resulting in increased carbon storage relative to those that flowered later. While the studies demonstrated significant connections between carbon dynamics and phenology, the broader implications of phenological changes across many biomes have not yet been thoroughly investigated.

Early flowering plants had extended growth seasons which enabled them to absorb and sequester carbon, therefore changing the general carbon balance of the ecosystem. Scholz et al., [45] demonstrated that longer growing seasons increase carbon uptake and storage for alpine grasslands. Blume-Werry et al., [4] also found that early-leafing trees in a forest start photosynthesising and storing carbon earlier in the season, that helps to contribute more generally to carbon sequestration than late-leafing trees. For deciduous forests in the northeastern United States, Putnam and Reich [38], found out that trees that leafed out sooner had more rates of photosynthesis and carbon absorption than those that leafed out later. The results show that the degree of carbon ecosystems can store is much influenced by timing of phenological events, thereby underscoring the vital function of phenology in regulating ecosystem carbon dynamics.

An extended growing season is expected to result in a higher GPP as plants have more time to photosynthesis and accumulate biomass (M.-W. Li et al., [30]. However, the link between GPP and the length of the growing season is complicated. While some ecosystems adapt to shorter periods by rapid CO₂ absorption during peak growth phases, some flourish with longer growing seasons [13]. Temperature fluctuations, water and nutrient availability, and biome-specific adaptations all influence GPP’s response to changes in the length of the growing season [31]. Furthermore, early start of growing season may be more susceptible to late frosts or other environmental stresses, which would reduce carbon sequestration in comparison to their later-starting counterparts [11].

**Fig. 1:**
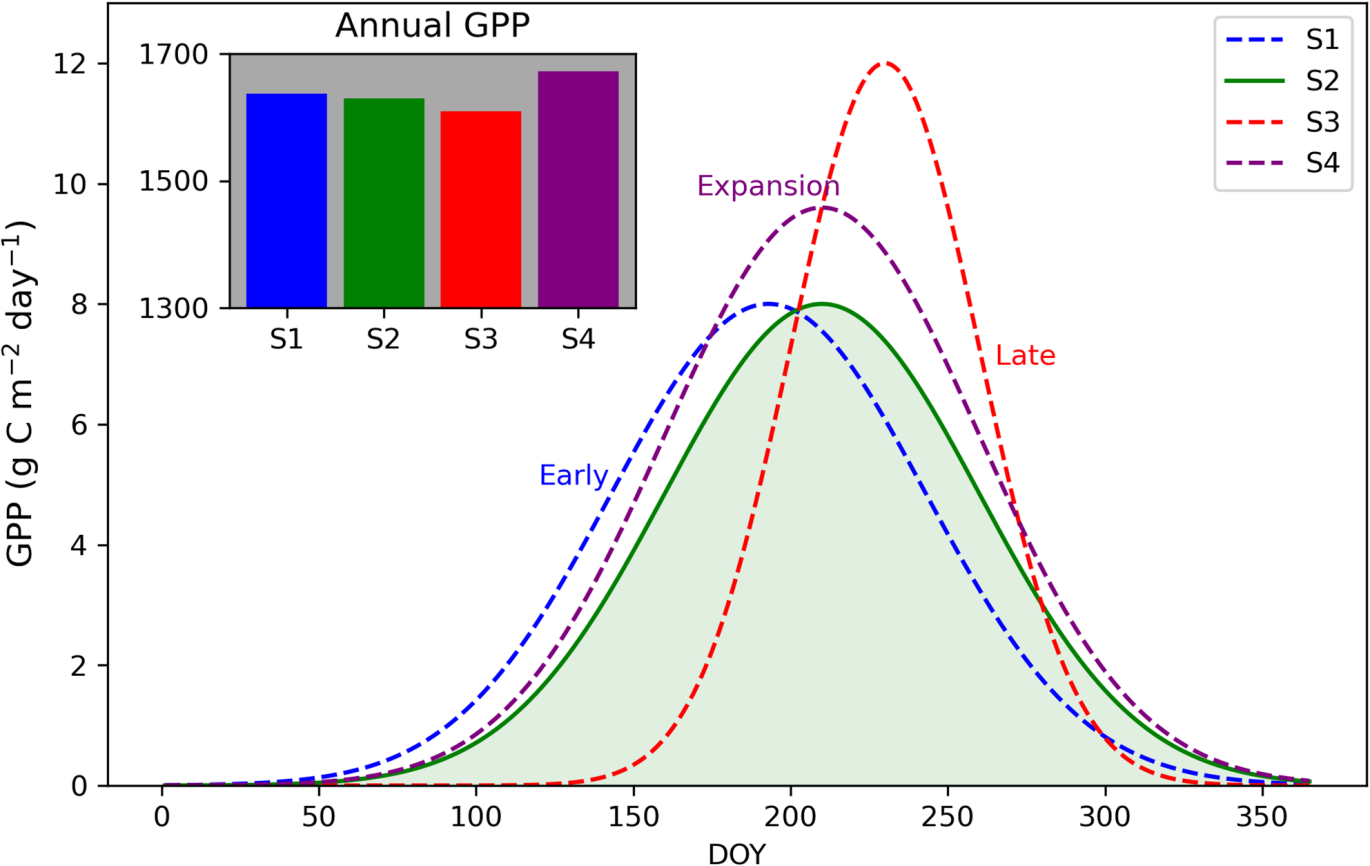
Schematic of possible plant phenology scenarios and annual GPP

Accurate estimate of future carbon dynamics is dependent on an understanding of these relationships as ecosystems adapt to changing climate conditions. Nevertheless, to the best of our knowledge, the exact quantitative effects of Length of Growing Season (LGS) on GPP remain insufficiently quantified, especially across diverse ecosystems. To illustrate this, we present three hypothetical schematic scenarios, Fig. 1, that capture potential changes in growing season dynamics for the northern hemisphere. These scenarios include: (1) an advanced growing season, (2) an expanded growing season, and (3) a delayed growing season, i.e., a short growing season with a high peak in productivity.

These scenarios illustrate the plausible consequences of various growing season dynamics on photosynthetic activity and carbon fluxes. Though the shape of the growing season curve differs, these fluctuations might not always clearly translate into significant annual GPP variations. This suggests that although the LGS and timing play a fundamental role in managing carbon sequestration, other variables including temperature, and precipitation, play a significant role in determining GPP. These scenarios raise an essential question: To what extent does the LGS control GPP across different biomes?

Although LGS and timing of SOS and EOS are key determinants of carbon uptake, there is still a gap in understanding how these dynamics influence annual GPP across diverse ecosystems. The main objective of this study is to quantify the impact of changes in the key phenological variables on GPP in diverse biomes using data from flux towers, particularly using the FLUXNET2015 dataset.

FLUXNET2015 provides globally distributed, standardized eddy‑covariance measurements and has been widely used to address ecological and carbon‑cycle questions. For example, Jung et al., [24] used the dataset to investigate how various environmental conditions regulate GPP, while [47] demonstrated the utility of FLUXNET eddy-covariance data for model training, validation, intercomparison, and fusion to improve large-scale GPP estimates. At the site level, long‑term flux tower records have provided insights into ecosystem carbon-water interactions, such as increasing GPP and water‑use efficiency in tropical savannas [20]. The extensive use of FLUXNET2015 across multiple scales highlights its suitability for evaluating phenological influences on ecosystem productivity.

Quantifying the relationship between phenological variability and GPP, would be useful in charactering the uncertainty related to phenological representation in carbon models. In Dynamic Global Vegetation Models (DGVMS), phenological processes are represented using a variety of methodologies, ranging from simple threshold-based models based on temperature or day length cues to more complex systems that consider plant functional characteristics and environmental variables. Simplified representations of phenological processes, such as temperature thresholds or photoperiod, fail to capture the complicated ecosystem-level reactions [8, 42]. This research can also give insights to improve phenology module simulations in DGVMs. This anticipated improvement can help in improving global carbon model estimations and obtaining more accurate estimates of ecosystems’ carbon storage.

## Materials and methods

### Study area and data used

In this study, we used daily aggregated GPP data from 132 sites included in the FLUXNET2015 dataset. The FLUXNET2015 dataset is a unique data source for studying ecosystem productivity as it includes data from a wide range of biomes worldwide offering opportunities for investigating global carbon cycling studies [16, 18]. We used the *GPP_DT_VUT_REF* variable, which represents daytime - partitioned GPP derived using the variable ustar threshold approach. This method accounts for temporal variability in turbulence and minimizes uncertainties associated with nighttime respiration, providing a robust estimate of ecosystem GPP [35]. For each site-year, annual GPP $\:\left(g\:C\:{m}^{-2}{yr}^{-1}\right)$ was computed as the sum of daily GPP values over the calendar year. Sites showing clear seasonal fluctuations in GPP were retained, whereas those with minimal or non-seasonal variability were excluded. Specifically, sites were removed if they (i) contained fewer than two complete years of observations, or (ii) lacked a distinct seasonal rise-and-fall pattern in GPP based on visual inspection of the seasonal trajectories, to ensure a reliable estimation of phenological metrics. For analyses requiring interannual anomalies (Sect. "Statistical analysis of growing season timing shifts"), we applied a stricter temporal criterion and only retained sites with four or more complete years of observations.

**Fig. 2:**
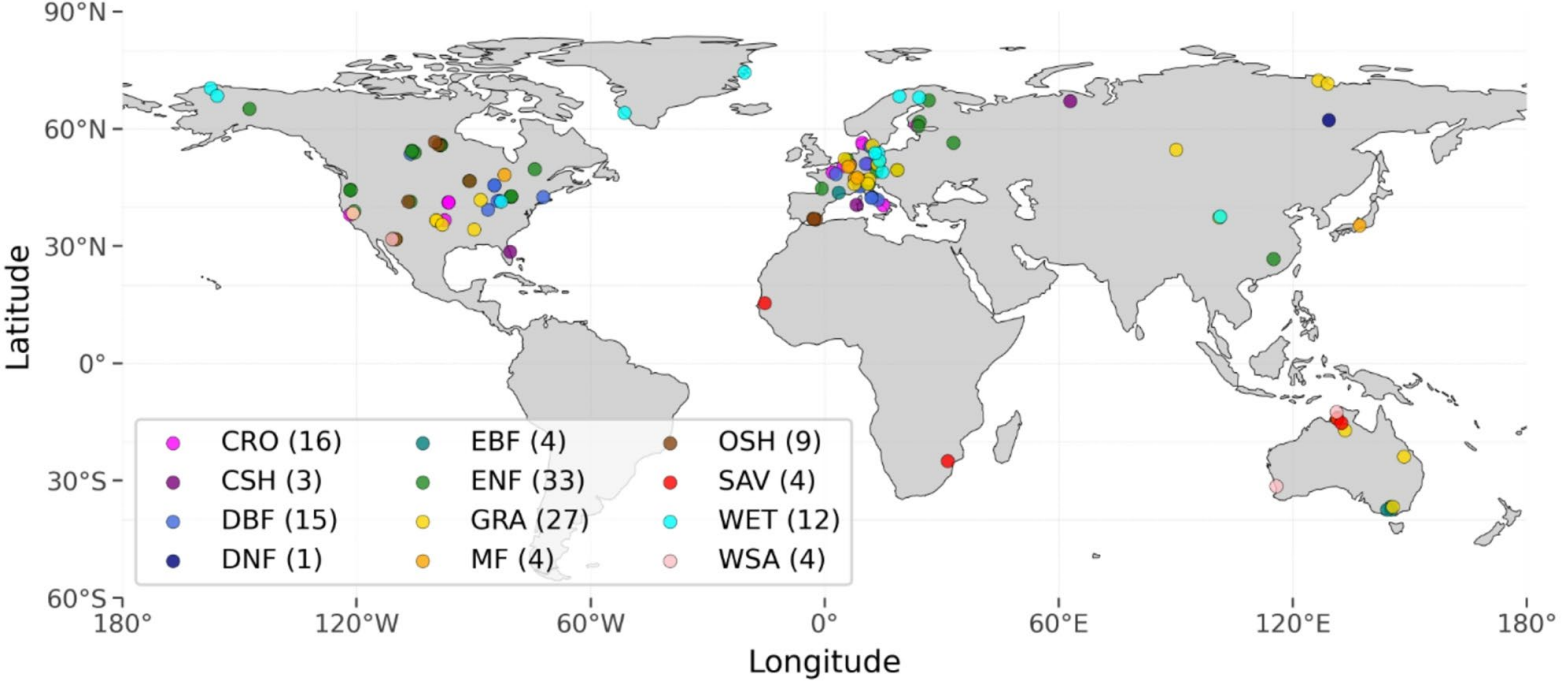
A map of the 132 selected FLUXNET2015 sites, color-coded by land cover class based on IGBP classifications. The figures in parentheses represent the count of sites within each IGBP category

The selected sites, shown in Fig. 2, span 12 land cover categories defined by the International Geosphere-Biosphere Program (IGBP): Croplands (CRO), Closed Shrublands (CSH), Deciduous Broadleaf Forests (DBF), Deciduous Needleleaf Forests (DNF), Evergreen Broadleaf Forests (EBF), Evergreen Needleleaf Forests (ENF), Grasslands (GRA), Mixed Forests (MF), Open Shrublands (OSH), Savannas (SAV), Wetlands (WET), and Woody Savannas (WSA). These sites were chosen to represent a diverse range of biomes with pronounced seasonal variations in GPP. In total 914 site-years of data were used in the analysis.

The FLUXNET2015 dataset is a result of collaboration across many networks, including AmeriFlux, ICOS, and AsiaFlux. The networks worked together to standardise data collection and processing methodologies, ensuring that the dataset contains high-quality, consistent data that can be used to conduct global environmental research [35]. With its applications in climatic variability, land use change, and carbon modelling FLUXNET2015 represents an essential resource for investigating GPP and other land-atmosphere carbon fluxes. The data is publicly available through the FLUXNET data portal (FLUXNET, 2021).

### Data smoothing

Fourier analysis is commonly used technique in phenology to smooth out temporal data, such as temperature. By breaking the data into its harmonic components, this method efficiently removes high-frequency noise while preserving the key patterns that define the underlying growing season.

The smoothed data is highly useful for identifying critical phenological dates, including the start and end of the growing season [1, 6, 33]. The Discrete Fourier Transform (DFT) is commonly employed to perform this analysis.

By applying the DFT we can extract the amplitude and time of the seasonal cycles in the temporal data as well as the phase and magnitude of every harmonic [44]. This is done by transforming the time-domain data into the frequency domain. The DFT of GPP is given by:

1 $$\:F\left(u\right)=\:\frac{1}{N}\sum\:_{t=0}^{N-1}GPP\left(t\right)*{e}^{-\frac{2\pi\:ut}{N}}$$

where 𝐹(𝑢) represents the Fourier transform of the GPP data at frequency $\:u,\:GPP\left(t\right)$ is the Gross Primary Productivity value at a time $\:t$ in the time series, $\:u\:$is the number of Fourier components, $\:t\:$is the time index in the time series, ranging from $\:0$ to $\:N-1$, where $\:N\:$is the total number of data points. The normalization factor ensures that the amplitude of the Fourier components is scaled correctly. Equation (1) is split into two parts: the real (cosine) part, $\:{F}_{C\left(u\right)}$, and the imaginary (sine) part, $\:{F}_{s\left(u\right)}$, given by:

2 $$\:{F}_{C\left(u\right)=\frac{1}{N}\sum\:_{t=0}^{N-1}GPP\left(t\right)*cos\left(\frac{2\pi\:ut}{N}\right)}$$

3 $$\:{F}_{S\left(u\right)=\frac{1}{N}\sum\:_{t=0}^{N-1}GPP\left(t\right)*sin\left(\frac{2\pi\:ut}{N}\right)}$$

Using these components Eqs. 2 and 3, we calculate the Fourier magnitude $\:\left({F}_{m}\right)\:$and phase $\:\left({F}_{p}\right)\:$ as follows:

4 $$\:{F}_{m\left(u\right)}=\sqrt{{{F}^{2}}_{C\left(u\right)}+{{F}^{2}}_{S\left(u\right)}}$$

and the phase $\:\left({F}_{p}\right):$

5 $$\:{F}_{p\left(u\right)}=atan2\left(\frac{{F}_{S\left(u\right)}}{{F}_{C\left(u\right)}}\right)$$

With the calculated parameters, we can reconstruct a smoothed version of the GPP data as follows:

6 $$\:{GPP}^{*}\left(t\right)=\:{F}_{m}\left(0\right)+\:\sum\:_{n=1}^{u}{F}_{m\left(n\right)}Cos\left(\frac{2\pi\:nt}{N}-{F}_{p\left(n\right)}\right)$$

where the term $\:{F}_{m}\left(0\right)$ represents the zero-frequency Fourier magnitude, which corresponds to the mean value of the GPP time-series data.

This method mainly focuses on lower-frequency harmonics, which reveal long-term patterns in GPP. By removing random, short-term variations we improve the time series to be cleaner of noises, thereby enabling more accurate extraction and assessment of phenological dates.

For the 132 selected sites we experiment with different harmonics using the DFT and finally determine the optimal number $\:u$ that best reflects the underlying seasonal changes. The number of harmonics ranged from 6 to 14, depending on the vegetation and seasonal conditions at each site. Sites with regular seasonal patterns required fewer harmonics, whereas sites with more irregular or complex vegetation patterns required more harmonics to effectively represent the seasonal dynamics and reduce noise. The variety in harmonics contributed to the preservation of each site’s distinct phenological features.

### Estimation of the start and end of season

**Fig. 3:**
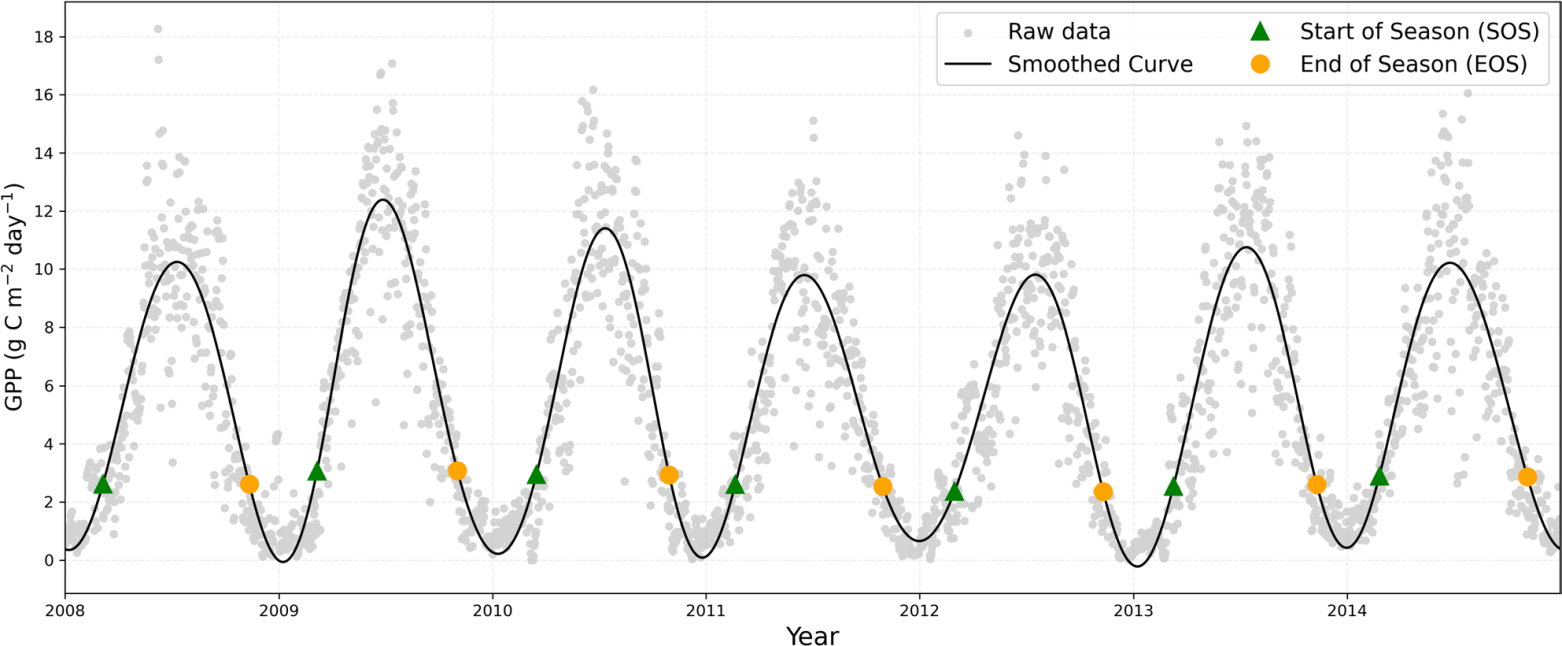
Detection of phenological events SOS and EOS from GPP data at site BE-Vie, Belgium. The figure shows raw GPP data, the smoothed curve from DFT analysis, and the SOS/EOS dates defined by slope-based percentiles, illustrating how the method captures the timing of phenological events

Threshold and change detection techniques are the main methods utilised within the land surface phenology research community to identify phenological events from time series of satellite-based vegetation index data. Threshold techniques, including fixed and dynamic thresholds, are popular because of their simplicity and practicality, especially in widely used tools like TIMESAT [23]. However, they lack underlying biophysical significance and particularly in places with complex seasonal cycles they are not suitable [3, 53]. On the contrary, change detection methods determine phenology days by directly identifying changing characteristics (e.g. slope) of the time series data. This is accomplished by either employing inflection points, which indicate changes in the direction of the curve’s curvature, or by identifying locations with high derivatives [7, 39, 54]. Compared to thresholds this method provides a process-linked assessment of the start and end of the growth season ([41] b). However, they miss smaller, gradual changes in ecosystems where transitions aren’t sharp [55] Fig. 3.

In this study, SOS and EOS for the GPP time series data are obtained using a slope-based approach. To determine the SOS, the slopes of the data before the peak of the growing season are first calculated i.e. identifying positive slope that signal the onset of greening. The SOS is then estimated as the date that corresponds to the 25th percentile of these positive slopes. On the other hand, the EOS is determined by selecting data after the peak and calculating the slopes. Negative slopes, indicating senescence, are identified, and the EOS is estimated as the 75th percentile of these negative slopes. Figure 3 illustrates this approach using the BE-Vie flux site, showing the raw GPP data, the smoothed DFT curve, and the derived SOS and EOS dates based on slope percentiles. This approach depends on the rate of change rather than a predetermined threshold, which makes it more appropriate for identifying phenological events across various ecosystems. The difference between the SOS and the EOS is used to calculate the length of the growing season. This method is repeated for the 132 flux towers, providing a flexible tool for phenometrics extraction.

### Statistical analysis of growing season timing shifts

To assess the impact of shifts in the timing of the growing season on GPP, we conducted an anomaly-based statistical analysis by calculating deviations in the start and end of the season $\:(\varDelta\:SOS\:$and$\:\:\varDelta\:EOS$) from their long-term means. By focusing specifically on $\:\varDelta\:SOS\:$or$\:\:\varDelta\:EOS$ rather than raw SOS/EOS values, we can isolate the effects of shifts in biome specific growing season timing on carbon uptake, offering a clearer understanding of how deviations from typical seasonal patterns can influence carbon flux and ecosystem productivity. Because reliable anomaly estimation requires sufficient interannual sampling, the anomaly analyses were restricted to sites with ≥ 4 complete years of data. Anomalous years were identified by calculating the standard deviation of SOS and EOS values over the study period, with years where SOS and EOS values deviated more than 1.5 standard deviations from the mean classified as anomalous. The anomalies were computed as the difference between the mean SOS and EOS for anomalous and normal years:

$$\begin{aligned}&\:\varDelta\:SOS=\:{{\mu\:}_{normal}-\mu\:}_{anomalous}\\&\:\mathrm{a}\mathrm{n}\mathrm{d}\:\varDelta\:EOS=\:{{\mu\:}_{normal}-\mu\:}_{anomalous}\end{aligned}$$

To examine the relationship between the anomalies and GPP, we performed simple linear regression for both $\:\varDelta\:SOS$ and Δ$\:EOS$:

$$\begin{aligned}&\:{\varDelta\:GPP}_{i}={\beta\:}_{0}+{\beta\:}_{1}{\varDelta\:SOS}_{i}+{\epsilon}_{i},\\&\:\mathrm{a}\mathrm{n}\mathrm{d}\:{\varDelta\:GPP}_{i}={\beta\:}_{0}+{\beta\:}_{2}{\varDelta\:EOS}_{i}+{\epsilon}_{i}\end{aligned}$$

where $\:{\beta\:}_{1}$​ and $\:{\beta\:}_{2}$​ ​ represent the regression coefficients for SOS and EOS anomalies, respectively. This regression approach isolates the effects of shifts in growing season timing on carbon uptake, offering a clearer understanding of how anomalous changes in seasonality influence GPP and carbon cycling.

## Results

### Influence of length of growing season (LGS) on GPP

**Fig. 4:**
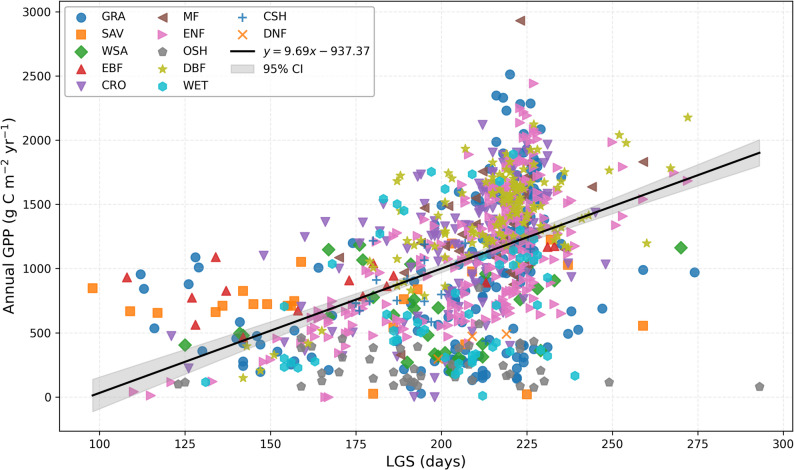
Regression plot illustrates the relationship between Length of Growing Season and Annual Gross Primary Productivity across 132 sites. Points color-coded by land cover type

Figure 4 depicts a modest positive association between LGS and annual GPP over all 132 sites, indicating that GPP increases by 9.6 g C m⁻² yr^−1^ for each additional day of LGS. The correlation coefficient $\:(r=0.48)\:$supports this moderate positive relationship.

Further, the influence of LGS on GPP connection was investigated using regression analysis across multiple land cover classes, and the results are reported in Table 1. LGS and GPP had a statistically significant positive relationship across six land cover classes: DBF $\:(r\:=\:0.68)$, ENF $\:(r\:=\:0.66),$ GRA $\:(r\:=\:0.39)$, CRO $\:(r\:=\:0.41),\:$EBF $\:(r\:=\:0.67),$ and MF$\:\:(r\:=\:0.41)$, though the strength of this relationship varied.

**Table 1:** Summary of Regression Analysis grouped by land cover class

IGBP Class	Equation	Correlation	Significance	
DBF	$\:Y=\:11.94x-1153.01$	0.7062	***	
ENF	$\:Y=\:13.23x-1637.57$	0.6666	***	
GRA	$\:Y=\:8.24x-703.07$	0.3936	***	
CRO	$\:Y=\:10.25x-923.31$	0.4729	***	
CSH	$\:Y=\:3.10x+270.31$	0.1915		
DNF	$\:Y=\:9.64x-1594.21$	0.9020		
EBF	$\:Y=\:2.98x+376.56$	0.5863	*	
MF	$\:Y=\:10.66x-932.69$	0.4114	**	
OSH	$\:Y=\:-0.05x+258.25$	−0.0102		
SAV	$\:Y=\:-0.00x+722.33$	−0.0002		
WET	$\:Y=\:4.81x-219.64$	0.2306		
WSA	$\:Y=\:2.95x+92.35$	0.2506		

Statistical significance is denoted by asterisks: ***p < 0.001, **p < 0.01, and *p < 0.05. Results without asterisks indicate that the LGS is not statistically significant predictor at the standard thresholds

**Fig. 5:**
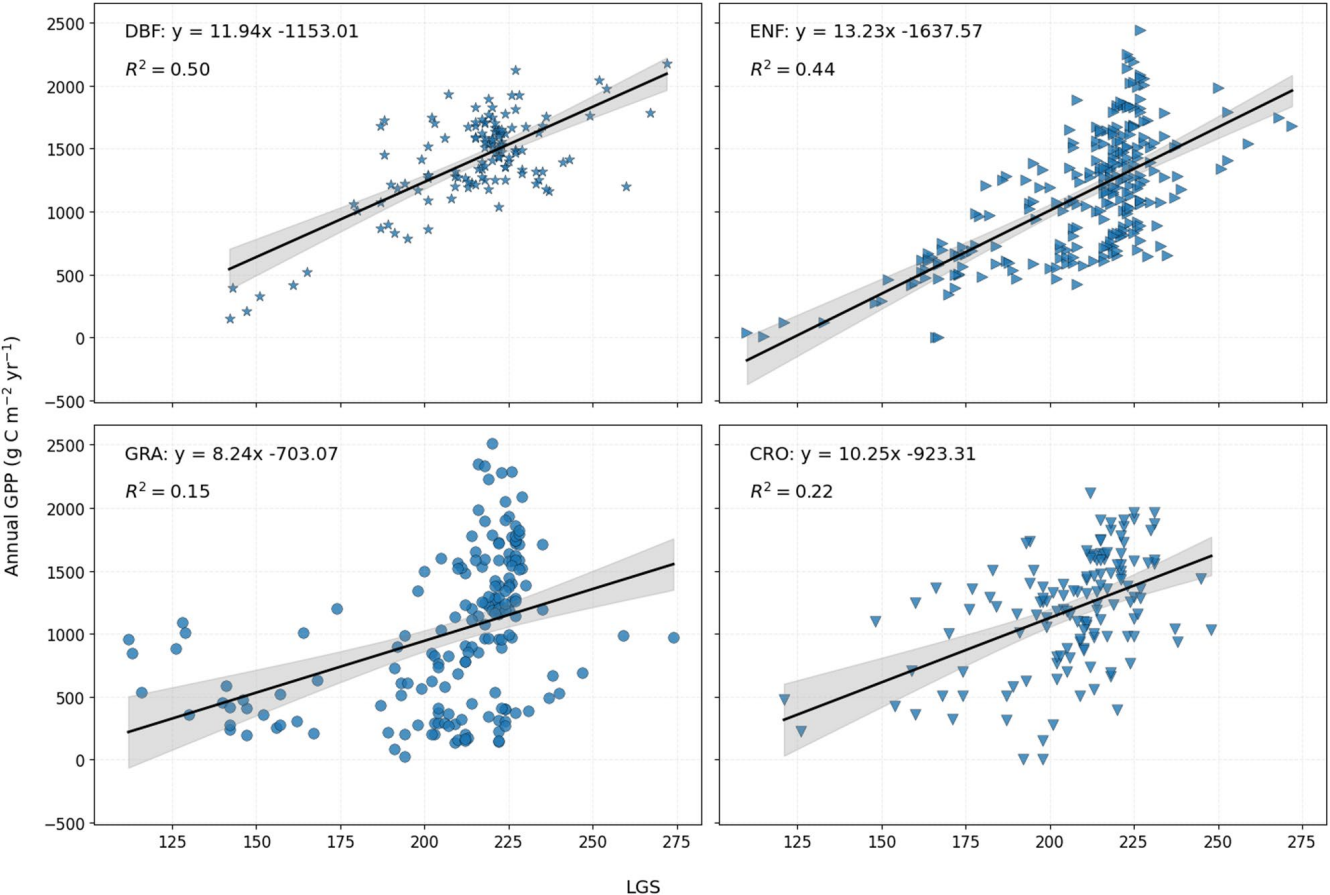
Regression plots showing the relationships between LGS and GPP for Deciduous Broadleaf Forest (DBF), Evergreen Needleleaf Forest (ENF), Grassland (GRA), and Cropland (CRO) land cover classes

Notably, DBF and ENF show strong positive correlations with LGS explaining over $\:50\%$ and $\:44\%$ of the variability in GPP in these environments respectively. On the other hand, GRA and CRO classes show modestly significant relationships with the coefficient of determination $\:0.15$ and $\:0.22$, respectively, suggesting that in these ecosystems other environmental variables could be more important in influencing GPP, Fig. 5.

### Impact of start of season anomalies $\:\left({\Delta}\mathrm{SOS}\right)$ on GPP

**Fig. 6:**
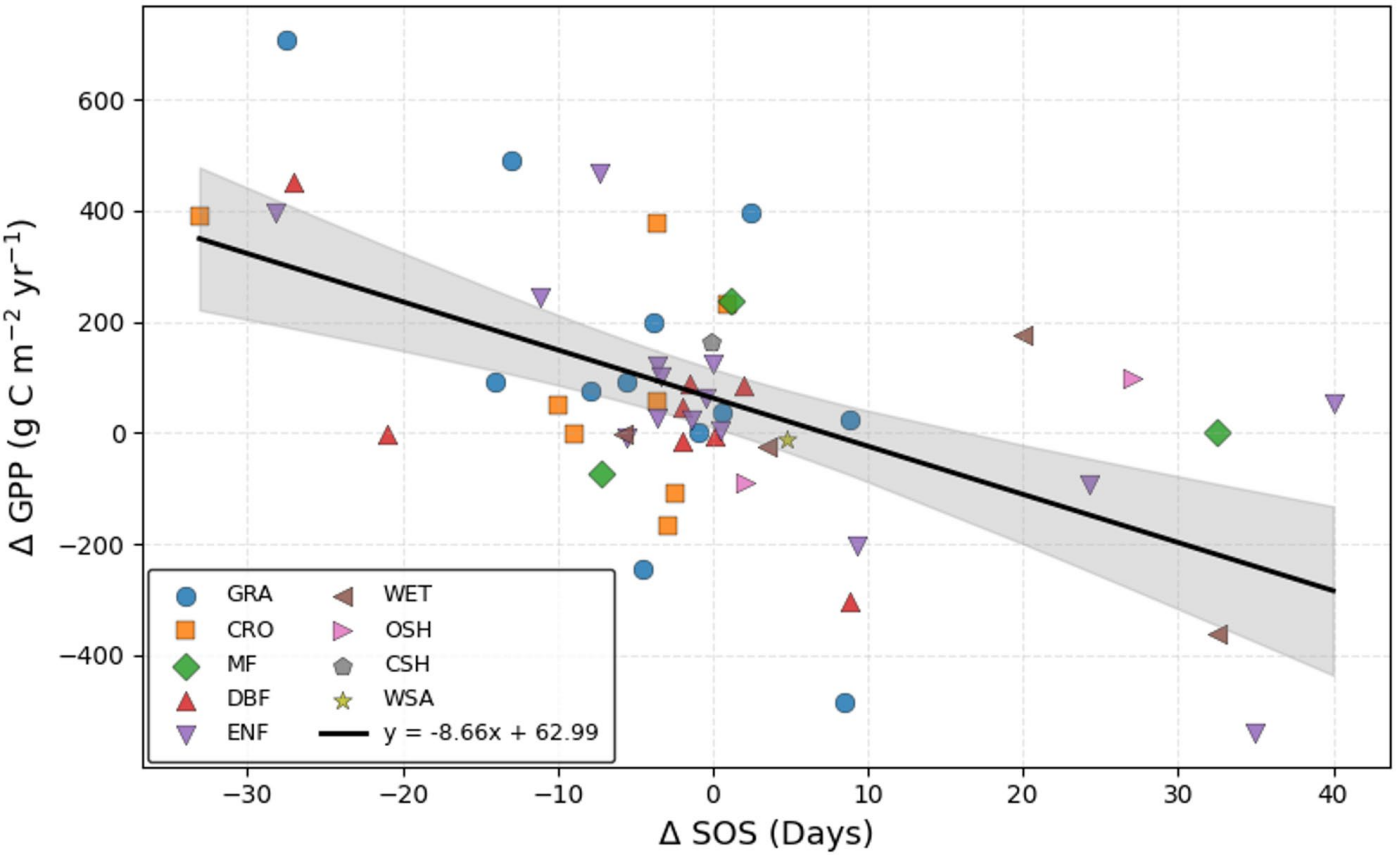
Regression analysis of ΔSOS vs. ΔGPP, with points color-coded by land cover type

A regression analysis was performed to examine the relationship between the start of season anomalies $\:\left(\varDelta\:SOS\right)$ and yearly GPP anomalies $\:\left(\varDelta\:GPP\right).$ Fig. 6 illustrates a significant negative association between $\:\varDelta\:SOS$ and $\:\varDelta\:GPP$ indicating that a postponement in the onset of the growing season $\:\left(\varDelta\:SOS\right)$ correlates with a decline in GPP. On average one day earlier start of season results in an increase in GPP of 8.66 g C m⁻² day^−1^ ($\:R^{2}\:=\:0.56)$.

**Fig. 7:**
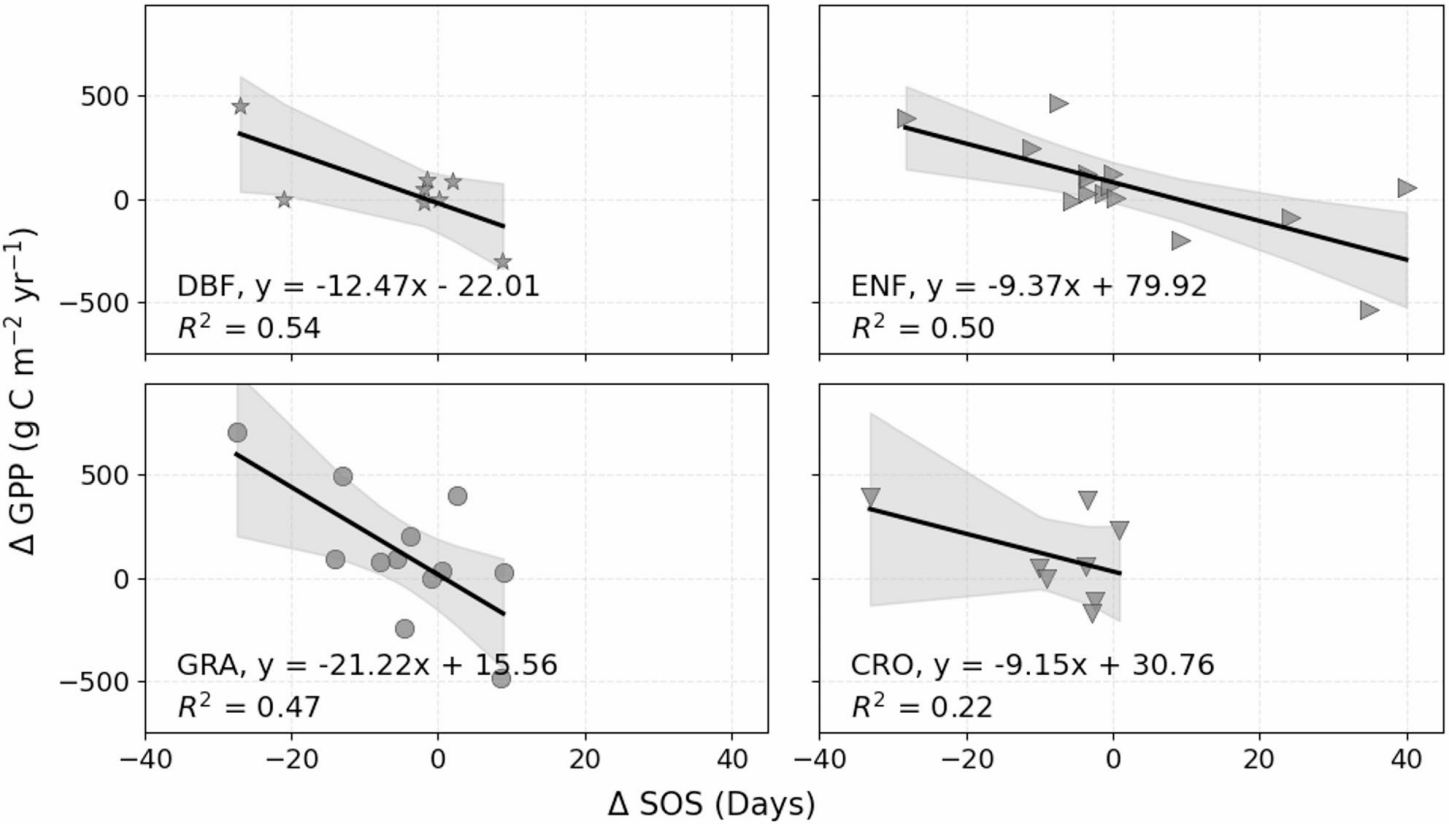
Regression analysis of ΔSOS vs. ΔGPP, for DBF, ENF, GRA, and CRO land cover classes

Biomes specific analysis reveals important notable variations. ENF, GRA and WET show a significant negative correlation between $\:\varDelta\:SOS\:$and $\:\varDelta\:GPP$. Figure 7 illustrates the significant negative correlations found in DBF, ENF and GRA, implying that timing of the growing season influences carbon absorption in these biomes with $\:R^{2}$ values of 0.54, 0.5 and $\:0.47$ respectively.

Conversely, CRO with $\:\left(\:\:{R}^{2}=\:0.22\right)$ do not show a statistically significant correlation. This suggests that this biome is less sensitive to changes in the start of the growing season due to other dominant environmental and anthropogenic factors. Furthermore, OSH, showed weak relationships highlighting the overriding effect of disturbances on productivity in these ecosystems.

### Impact of end of season anomalies ($\:{\Delta\:}\mathrm{EOS}$) on GPP

**Fig. 8:**
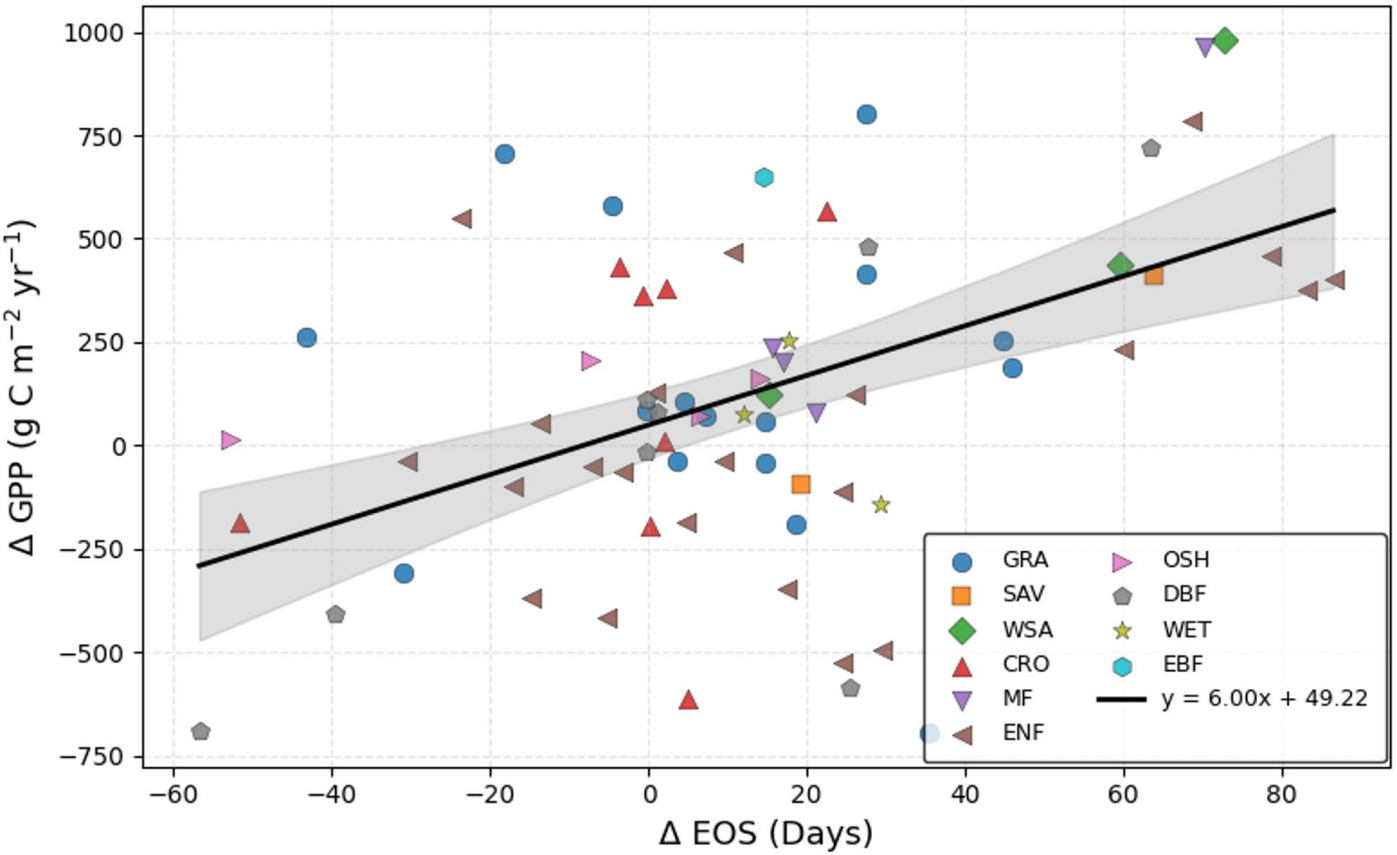
Regression analysis of ΔSOS vs. ΔGPP, with points color-coded by land cover type

A regression analysis was performed to evaluate the relationship between $\:\varDelta\:EOS$ and $\:\varDelta\:GPP$. As shown in Fig. 8, a moderate positive correlation between $\:\varDelta\:EOS$ and $\:\varDelta\:GPP$ $\:(R^{2}\:=\:0.38)$ exits indicating that one day delay in $\:\varDelta\:EOS\:$results in an increase of 6 g C m⁻² day^−1^ in GPP. Although the relationship is statistically significant, it is weaker than that observed between $\:\varDelta\:SOS$ and $\:\varDelta\:GPP$.

**Fig. 9:**
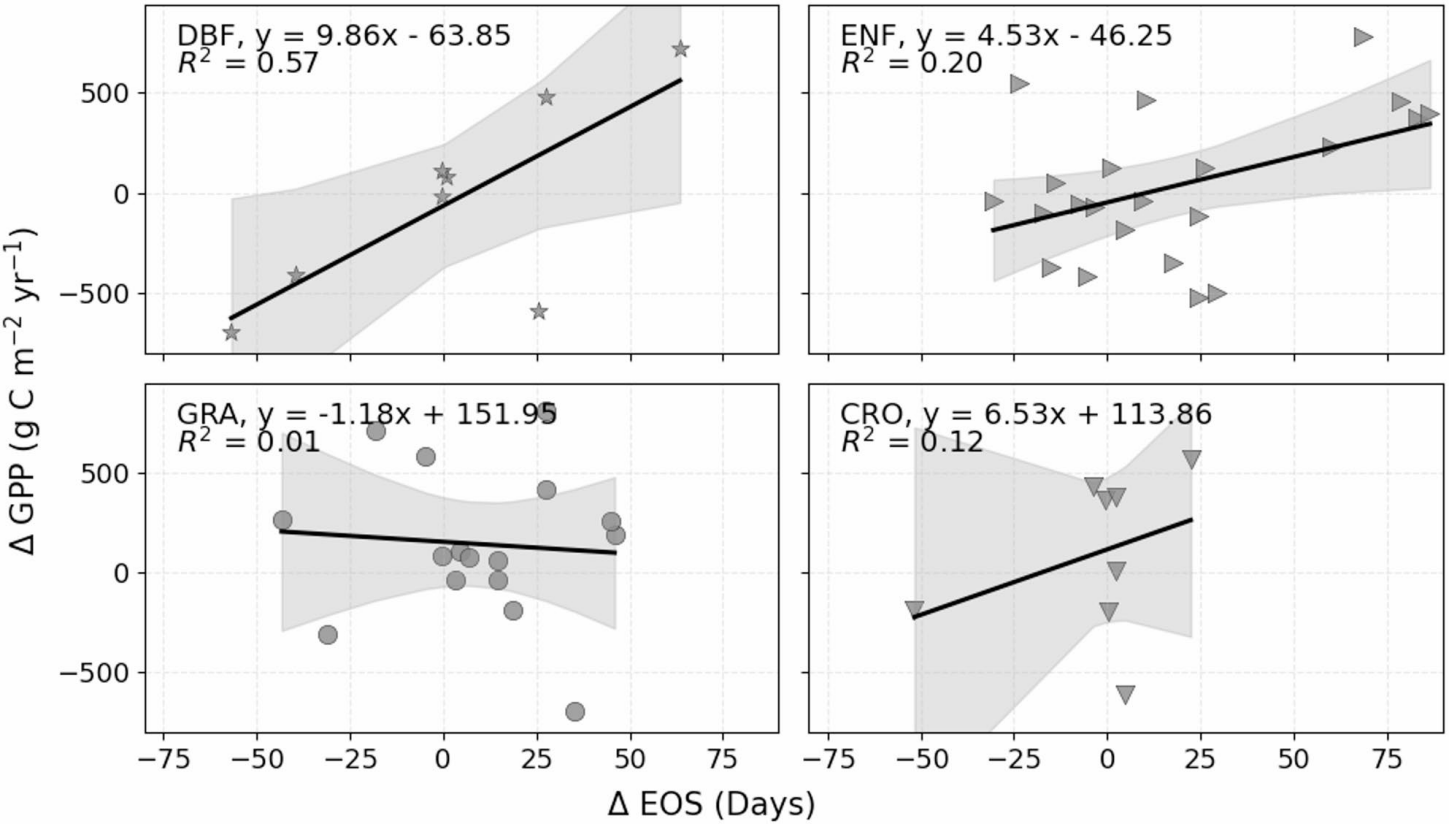
Regression analysis of ΔEOS vs. ΔGPP, for DBF, ENF, GRA, and CRO land cover classes

Biome-specific analyses, Fig. 9, indicate that, among the biomes, the temperate biome DBF exhibits significant positive correlations between $\:\varDelta\:EOS$ and $\:\varDelta\:GPP$ with $\:(R^{2}\:=\:0.57,)$.

In contrast, ENF, GRA, and CRO exhibit weak correlations. With ENF and GRA, they have very low $\:R^{2}\:$values 0.01 and 0.2 respectively, and CRO’s $\:R^{2}\:$value is also low 0.12, with the weak relationships attributed to factors like water availability in GRA and agricultural management in CRO.

### Linking phenology across biomes to carbon uptake

**Fig. 10:**
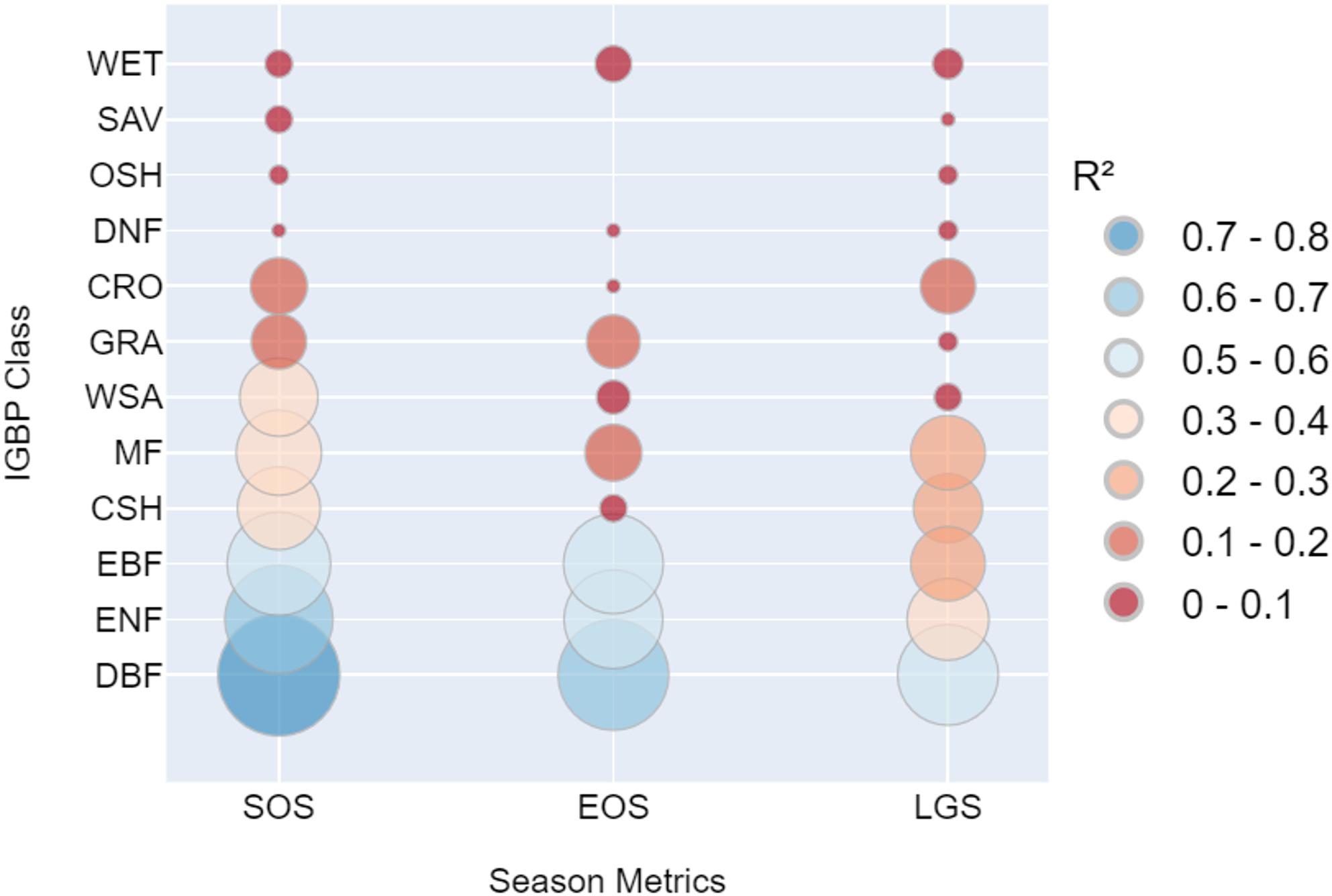
Season metric vs. growing season GPP

Figure 10 provides a visual representation of the intensity and direction of the relationships between SOS, EOS, LGS, and GPP across various biomes. Earlier occurrence of SOS shows a significant correlation with increased GPP in forest biomes, particularly in DBF $\:(R^{2}\:=\:0.78),$ ENF $\:(R^{2}\:=\:0.61)$, and EBF $\:(R^{2}\:=\:0.56).$ Conversely, lower correlations observed in GRA, CRO, and WET $\:(R^{2}\:<\:0.2)$ indicating that additional factors may have a more significant influence in these systems.

The influence of EOS on GPP is notable for DBF $\:(R^{2}\:=\:0.53)$ and ENF $\:(R^{2}\:=\:0.35),$ as the postponement of senescence improves carbon uptake. Nonetheless, the impact of EOS is minimal in most other biomes, exhibiting weak or negligible correlations in GRA, CRO, and WET $\:(R^{2}\:<\:0.1)$, which suggests a reduced sensitivity to late-season shifts. Also, we found a positive correlation between LGS and GPP in various forest types, specifically DBF $\:(R^{2}\:=\:0.64),$ ENF $\:(R^{2}\:=\:0.51)$, and EBF $\:(R^{2}\:=\:0.52),$ thereby reinforcing the hypothesis that extended growing seasons may contribute to increased productivity. Non-forest biomes, such as GRA, CRO, and WET, demonstrate low $\:R^{2}\:$ values $\:(R^{2}\:<\:0.2),$ highlighting the dependence of this relationship on the specific biome.

## Discussions

Using data from the FLUXNET2015 network, this study examined the relationships between phenological changes and carbon dynamics across various biomes. The findings emphasise the differential impact of changes in the LGS, SOS, and EOS on GPP particularly in temperate, boreal, and tropical biomes. Although previous studies have examined phenological dynamics using FLUXNET data, the approach adopted here integrates high-temporal-resolution flux tower observations across multiple biomes, which supports the generalisability of our findings. To further evaluate the spatial generalisability of flux tower-based phenology, Fluxnet2015 SOS was compared with satellite-derived SOS from MODIS (Greenup_1, MCD12Q2.006) (figure S1). MODIS SOS lagged the GPP-derived SOS by ~ 35 days, indicating that it reflects mid-greenup rather than the true onset of photosynthesis. Despite this offset, MODIS preserved relative spatial and interannual patterns, supporting the use of GPP-derived SOS to capture spatial phenological variability. These results contribute to an understanding of how phenological shifts affect carbon dynamics and provide insights into improving carbon modeling in diverse ecosystems.

### The role of LGS on GPP

The findings highlight the impact of LGS on GPP, illustrating that a prolonged growing season enhances carbon uptake. While the relationship between LGS and GPP is statistically significant, the coefficient of determination ($\:{R}^{2}\:=\:0.23$) suggests that LGS accounts for only 23% of the variability in GPP. This indicates that temperature, rainfall, soil moisture, and other physiological processes specific to different species significantly influence GPP dynamics [46].

The impact of LGS on GPP exhibited significant variability depending on biomes. Deciduous broadleaf forests and Evergreen needleleaf forests showed stronger positive correlations than the other biomes. The positive association in these biomes is due to the relatively stable and predictable growth patterns in these ecosystems. A stable and predictable season leads to greater carbon assimilation, because plants can efficiently utilize resources and allocate energy for growth in comparison to fluctuating season that causes stress by disrupting photosynthesis and carbon assimilation. These biomes are dominated by plant species exhibiting established seasonal growth cycles, wherein the duration of the growing season significantly influences carbon absorption [48, 57]. On the other hand, closed shrublands, open shrublands, Savannas, wetlands, and woody savannas were the biome groups that exhibited less correlation and had non-significant relationship between GPP and LGS. The less obvious relationship between LGS and GPP in these ecosystems is revealed by the fact that temperature, water availability, and certain environmental factors (such as fire or drought in savannas) primarily affect productivity [5, 46] than the phenological timing. Moderate positive connections were seen in croplands; yet human activities can complicate the relationship. Agricultural practices such as crop variety and irrigation provide more variation in croplands, thereby complicating the link between LGS and GPP [27].

### The role of SOS on GPP

Our results indicate that the reduction in GPP is a consequence of the shortening of the window of favourable early season growing conditions due to delays in SOS. This effect is most pronounced in ecosystems where plant development is initiated by early-season temperature and light conditions. These results align with prior research indicating that SOS plays a significant role in carbon flux, particularly in temperate biomes where early-season climatic conditions are essential for photosynthesis and production [13].

We found that carbon sequestration in Deciduous Broadleaf Forests, Evergreen Needleleaf Forests and Grasslands is significantly influenced by early-season conditions, which is why the sensitivity to SOS timing is high. The negative correlation observed in these biomes suggests that delays in SOS disrupt the early growth period, resulting in a substantial reduction in carbon assimilation. The negative SOS-GPP sensitivity we observe, especially in grasslands aligns with Li et al., [29], who showed that seasonal temperature and moisture regimes tightly control grassland productivity in Inner Mongolia; by delaying the onset of favourable early‑season conditions (later SOS), hence the productive window is truncated, reducing seasonal/annual carbon assimilation. Likewise, coniferous forests such as Evergreen Needleleaf Forests are susceptible to delays in SOS, as their carbon absorption is significantly influenced by the availability of light and temperature during the early season [15]. In croplands, the relationships observed were not statistically significant. The reduced sensitivity to changes in SOS suggest that additional environmental elements, like nutrient composition and water availability, play a more significant role in influencing productivity [43]. Human interventions such as crop variety selection, irrigation practices, and fertilisation techniques mitigate the impacts of SOS anomalies in agricultural systems thereby indicating these ecosystems less vulnerable to shifts in phenology [59]. Open shrublands, which are prone to drought and fire, showed poor associations and showed that disturbances, not phenological shifts, drive productivity in these ecosystems. Prieto et al., [37] demonstrated that drought spells severely lower shrubland productivity, overshadowing the role of SOS.

These findings also provide importance of accurate characterisation of vegetation phenology in Dynamic Global Vegetation Models (DGVMs). Current DGVMs employ a range of climate- and empirically based approaches to simulate phenological transitions [10], while our results highlight strong and biome-dependent sensitivities of GPP to SOS timing. This evidence provides context for improving representations of phenological processes in DGVMs. An overview of how major DGVMs currently represent phenology is provided in supplementary material (table S3).

### The role of EOS on GPP

The findings from our investigation reveal that changes in the EOS exert a moderate influence on carbon dynamics within ecosystems, with notable positive correlations identified in Deciduous broadleaf forests and Evergreen needleleaf forests. These biomes have a clear seasonal growth pattern, where a change in temperature and precipitation affect the timing of both the start and end of the growing season [58]. Late-season dynamics affect these ecosystems because their productivity is more tightly coupled to temperature and photoperiod [28]. As temperatures begin to drop in the late season, these biomes experience a decline in photosynthetic activity, which have a direct effect on the amount of carbon assimilated. This finding is consistent with previous studies that have highlighted the role of EOS conditions in temperate forest ecosystems, where the LGS and its timing influence carbon flux [58]. Relationships in crops, wetlands, and grasslands were not significant. Productivity in these biomes is mainly affected by the availability of water and precipitation during the growing season, then by the timing of EOS. In wetlands and grasslands, the amount of water significantly influences carbon flux more than the timing of EOS. Similarly, in Croplands, agricultural practices such as irrigation, crop varieties, and fertilization significantly affect more than the EOS anomalies.

While EOS extensions have increased photosynthesis in some biomes, their impact on global carbon dynamics is not as pronounced as that of SOS. This is largely related to the fact that plants have already reached their maximum production, hence ecosystems are less sensitive to late season variations. As the growing season draws to a close, the accumulation of biomass generally begins to decelerate, with environmental factors such as temperature and light exerting diminished influence. The reduced sensitivity to EOS shifts aligns with the observations made by [52], who indicated that late-season variations exert a relatively small influence on carbon assimilation when contrasted with early-season dynamics.

### Global upscaling and carbon sequestration potential

The global implications of our findings are substantial, yet they require careful interpretation. Our site-level analysis indicates that each additional day of the growing season is associated with an average increase of 9.6 g C m⁻² yr^− 1^ in annual GPP (with standard error 0.57 g C m⁻² yr^− 1^). If this relationship were hypothetically applied uniformly to the global vegetated land area (~ 107.8 million km², estimated from the 2023 MODIS, MCD12Q1.061 land cover product), it suggests a potential increase in global carbon uptake of approximately 1.035 Gt C yr^− 1^ per additional day of LGS (uncertainty range of: 0.98–1.11 Gt C yr^− 1^, propagated from the standard error of the slope). The global vegetated area was calculated in Google Earth Engine from pixel-level surface area (m^2^) in the native MODIS sinusoidal projection which accounts for the varying pixel sizes with latitude.

Similarly, based on our analysis, a one-day advancement in the start of season anomaly was associated with an average increase of 8.66 g C m⁻² yr^− 1^ in annual GPP (with standard error 1.77 g C m⁻² yr^− 1^). When scaled to the global vegetated land area this corresponds to a potential increase in global carbon uptake of approximately 0.93 Gt C yr^− 1^ with uncertainty range of (0.74–1.12 Gt C yr^− 1^). End-of-season anomalies exhibited a limited effect on GPP, where a one-day delay led to an average increase of 6.00 g C m⁻² yr^− 1^ GPP (with standard error 1.17 g C m⁻² day^− 1^). When hypothetically applied to the global vegetated land surface, this results in an enhanced carbon uptake of approximately 0.65 Gt C yr^− 1^ with uncertainty range of (0.52–0.77 Gt C yr^− 1^). The findings indicate that early season phenological shifts influence ecosystem productivity more than the LGS and EOS.

For context, compared to the recent Global GPP estimate of ~ 120 Pg C/yr [15], the combined first order effect of phenological shifts of 2.62 $\:\pm\:$ 0.24 Gt C yr^− 1^ (summing LGS, SOS and EOS) represents approximately 2.2% $\:\pm\:$ 0.2% of the total photosynthetic carbon uptake, underscoring the role of phenological shifts in regulating global carbon dynamics. However, this estimate does not account for concurrent changes in ecosystem respiration (RE), which may offset part of the GPP gains. For instance [25], showed that while both GPP and RE increased in response to earlier springs and extended seasons, the increase in GPP exceeded that of RE, resulting in a positive net carbon uptake. Future studies should incorporate RE to better constrain the carbon balance implications of phenological shifts.

It is important to note that this upscaling assumes uniform responses across all biomes, ignoring regional differences in climate sensitivity, ecosystem type, and land use. Furthermore, the uncertainty ranges presented here reflect only the variability in the site-level relationships and do not include additional sources such as land-cover classification uncertainty or regression intercept uncertainty. Thus, this analysis should be interpreted as a first-order, illustrative approximation rather than a predictive global estimate. This upscaling result gives us an idea of the scale at which phenological changes may impact global carbon cycles; however, it makes the simplistic assumption that responses will be uniform throughout all biomes, ignoring regional differences in ecosystems, climatic sensitivity, and land uses. Nonetheless, the research highlights that even little changes in the growing season’s timing might have a significant impact on the absorption of carbon. We restate that this upscaling procedure should be viewed as a first-order theoretical approximation rather than a predictive estimate, despite the obvious global implications.

### Limitations

Although our research highlights the relationship between GPP and phenology metrics, it should be mentioned that certain limitations exist. First, the research made use of mainly a limited number of flux tower sites from the FLUXNET2015 dataset, which might not equally encompass the diversity of global ecosystems particularly in underrepresented regions, specifically biomes in Asia and Africa are underrepresented. Moreover, results in certain biomes might have been impacted by site-specific factors not especially considered, including land management, disturbance history, and type of soil. In addition, the investigation concentrated on broad phenological events, neglecting the impact of more intricate factors, including species-specific responses, soil properties, and the impact of extreme weather events or disturbances.

Future research should focus and provide more precise insights into the impact of climate-induced changes in phenology on global carbon sequestration by addressing these complexities. Also, future studies should focus on combining flux tower data with satellite-based remote sensing to enhance the temporal and spatial resolution of phenological monitoring. In addition, combining this observational data with outputs from DGVMs will help us better understand how phenological fluctuations affect global carbon cycles. Additionally, while climate drivers such as temperature, precipitation, and vapor pressure deficit can influence phenology, an analysis of these covariates was beyond the scope of the present study and is suggested as a direction for future research.

## Conclusions

This paper investigates the influence of phenological parameters SOS, EOS, and LGS on carbon uptake across several biomes using data from 132 FLUXNET2015 flux tower sites. The findings reveal that delays in SOS significantly reduce GPP, particularly in grasslands and temperate forests, which are sensitive to early-season conditions. The results showed that EOS has a lower influence on GPP, compared to SOS emphasising the importance of early-season dynamics in controlling carbon uptake.

A significant positive correlation between LGS and GPP at all sites were obtained, though LGS only explained 23% of the variance in GPP, indicating that other environmental variables had a considerable influence on productivity. Biome-specific studies indicated that Deciduous broadleaf and Evergreen needleleaf forests had stronger connections between LGS and GPP, whereas closed shrublands, open shrublands, savannas, wetlands, and woody savannas had weaker connections.

## Supplementary information

Supplementary material 1

## Data Availability

The dataset used in this study was extracted from the FLUXNET2015 data portal ([https://fluxnet.org/data/fluxnet2015-dataset/]. Additional information related to this publication is contained in Supporting Information S1.
