# Supplementary material for: The impact of phenological shifts on carbon uptake across major terrestrial biomes
Source: Carbon Balance Manag. 2026 May 6;21:95. doi: 10.1186/s13021-026-00450-4 (PMC13317297; doi:10.1186/s13021-026-00450-4)
Supplement: Supplementary file 1 — Supplementary material 1 [file 13021_2026_450_MOESM1_ESM.pdf]

Supplementary Document

Section 1. This section provides a complete list of the flux tower sites included in the analysis. These sites were selected from the FLUXNET2015 dataset and represent a range of terrestrial biomes across the globe.

Table S1. Full list of flux tower sites used in this study

No	Site	Latitude	Longitude	IGBP	No	Site	Latitude	Longitude	IGBP
1	AT-Neu	47.116	11.317	GRA	67	CN-HaM	37.37	101.18	GRA
2	AU-DaP	-14.063	131.318	GRA	68	CZ-BK1	49.502	18.536	ENF
3	AU-DaS	-14.159	131.388	SAV	69	CZ-BK2	49.494	18.542	GRA
4	AU-Dry	-15.258	132.370	SAV	70	CZ-wet	49.024	14.770	WET
5	AU-Emr	-23.858	148.474	GRA	71	DE-Akm	53.866	13.68	WET
6	AU-Gin	-31.376	115.713	WSA	72	DE-Geb	51.099	10.914	CRO
7	AU-How	-12.494	131.152	WSA	73	DE-Gri	50.95	13.512	GRA
8	AU-Rig	-36.649	145.575	GRA	74	DE-Hai	51.079	10.452	DBF
9	AU-Stp	-17.150	133.350	GRA	75	DE-Kli	50.893	13.522	CRO
10	AU-Wac	-37.425	145.187	EBF	76	DE-Lkb	49.099	13.304	ENF
11	AU-Whr	-36.673	145.029	EBF	77	DE-Obe	50.786	13.721	ENF
12	AU-Wom	-37.422	144.094	EBF	78	DE-RuR	50.621	6.3041	GRA
13	BE-Lon	50.551	4.746	CRO	79	DE-RuS	50.865	6.4471	CRO
14	BE-Vie	50.304	5.998	MF	80	DE-Seh	50.870	6.4497	CRO
15	CA-Gro	48.216	-82.155	MF	81	DE-Spw	51.892	14.033	WET
16	CA-Man	55.879	-98.480	ENF	82	DE-Tha	50.962	13.565	ENF
17	CA-NS1	55.879	-98.483	ENF	83	DE-Zrk	53.875	12.889	WET
18	CA-NS2	55.905	-98.524	ENF	84	DK-Eng	55.690	12.191	GRA
19	CA-NS3	55.911	-98.382	ENF	85	DK-Fou	56.48	9.587	CRO
20	CA-NS5	55.8631	-98.485	ENF	86	DK-Sor	55.485	11.644	DBF
21	CA-NS6	55.9167	-98.964	OSH	87	ES-Amo	36.833	-2.252	OSH
22	CA-NS7	56.6358	-99.948	OSH	88	ES-LgS	37.097	-2.965	OSH
23	CA-Oas	53.6289	-106.197	DBF	89	ES-LJu	36.926	-2.752	OSH
24	CA-Obs	53.9872	-105.117	ENF	90	FI-Hyy	61.847	24.294	ENF
25	CA-Qfo	49.6925	-74.3421	ENF	91	FI-Jok	60.898	23.513	CRO
26	CA-SF1	54.485	-105.817	ENF	92	FI-Let	60.641	23.959	ENF
27	CA-SF2	54.2539	-105.877	ENF	93	FI-Lom	67.997	24.209	WET
28	CA-TP2	42.7744	-80.4588	ENF	94	FI-Sod	67.362	26.638	ENF
29	CA-TP3	42.7068	-80.3483	ENF	95	FR-Fon	48.476	2.7801	DBF
30	CA-TP4	42.7102	-80.3574	ENF	96	FR-Gri	48.844	1.9519	CRO
31	CA-TPD	42.6353	-80.5577	DBF	97	FR-LBr	44.717	-0.769	ENF
32	CH-Cha	47.2102	8.4104	GRA	98	FR-Pue	43.741	3.595	EBF
33	CH-Dav	46.8153	9.8559	ENF	99	GL-NuF	64.130	-51.386	WET
34	CH-Fru	47.1158	8.5378	GRA	100	GL-ZaF	74.481	-20.554	WET
35	CH-Lae	47.4783	8.3644	MF	101	IT-BCi	40.523	14.957	CRO
36	CH-Oe1	47.2858	7.7319	GRA	102	IT-Col	41.849	13.588	DBF
37	CH-Oe2	47.2864	7.7337	CRO	103	IT-Isp	45.812	8.633	DBF
38	CN-Qia	26.7414	115.0581	ENF	104	IT-Lav	45.956	11.281	ENF
39	CN-Ha2	37.6086	101.3269	WET	105	IT-MBo	46.014	11.045	GRA
40	IT-Noe	40.606	8.151	CSH	106	US-Ivo	68.486	-155.75	WET
41	IT-PT1	45.200	9.061	DBF	107	US-KS2	28.608	-80.671	CSH
42	IT-Ro1	42.408	11.93	DBF	108	US-Me2	44.452	-121.558	ENF
43	IT-Ro2	42.390	11.920	DBF	109	US-Me3	44.315	-121.607	ENF
44	IT-Tor	45.844	7.578	GRA	110	US-Me5	44.437	-121.566	ENF
45	JP-SMF	35.261	137.078	MF	111	US-Me6	44.323	-121.607	ENF
46	NL-Hor	52.240	5.071	GRA	112	US-MMS	39.323	-86.413	DBF
47	NL-Loo	52.166	5.743	ENF	113	US-Ne1	41.165	-96.476	CRO
48	RU-Fyo	56.461	32.922	ENF	114	US-Ne2	41.164	-96.470	CRO
49	RU-Ha1	54.725	90.002	GRA	115	US-Ne3	41.179	-96.439	CRO
50	RU-Sam	72.373	126.495	GRA	1116	US-Oho	41.55	-83.8438	DBF
51	RU-SkP	62.255	129.168	DNF	1117	US-Prr	65.123	-147.487	ENF
52	RU-Tks	71.594	128.887	GRA	118	US-Sta	41.396	-106.802	OSH
53	RU-Vrk	67.054	62.940	CSH	119	US-SRC	31.908	-110.839	OSH
54	SE-St1	68.354	19.050	WET	120	US-SRG	31.789	-110.827	GRA
55	SN-Dhr	15.402	-15.432	SAV	121	US-SRM	31.821	-110.866	WSA
56	US-AR1	36.426	-99.42	GRA	122	US-Ton	38.4309	-120.966	WSA
57	US-AR2	36.635	-99.597	GRA	123	US-Twt	38.1087	-121.653	CRO
58	US-ARb	35.549	-98.040	GRA	124	US-UMB	45.5598	-84.713	DBF
59	US-ARM	36.605	-97.488	CRO	125	US-UMd	45.5625	-84.697	DBF
60	US-Atq	70.469	-157.40	WET	126	US-Var	38.4133	-120.950	GRA
61	US-Blo	38.895	-120.63	ENF	127	US-Wi7	46.6491	-91.069	OSH
62	US-CRT	41.628	-83.347	CRO	128	US-Whs	31.7438	-110.052	OSH
63	US-GBT	41.365	-106.239	ENF	129	US-Wi4	46.7393	-91.166	ENF
64	US-Goo	34.254	-89.873	GRA	130	US-Wkg	31.7365	-109.941	GRA
65	US-Ha1	42.5378	-72.1715	DBF	131	US-WPT	41.4646	-82.9962	WET
66	US-IB2	41.8406	-88.241	GRA	132	ZA-Kru	-25.019	31.4969	SAV

Section 2. Global vegetated land area

To support the global extrapolation of our results, we estimated the areal extent of major vegetated land cover types using the MODIS Land Cover Type 1 product (MCD12Q1.061, 2023), which applies the International Geosphere-Biosphere Programme (IGBP) classification scheme. The global vegetated area provides context for calculating potential carbon uptake if observed site-level relationships between phenology and GPP were extended to all vegetated land surfaces based on the vegetation specific relationship between SOS and annual GPP.

Table S2. Global vegetated land area by MODIS IGBP land cover class

No	*Land cover type class*	*Area in km²*
1	Evergreen Needleleaf Forests	2,430,041.15
2	Evergreen Broadleaf Forests	11,965,559.90
3	Deciduous Needleleaf Forests	384,503.46
4	Deciduous Broadleaf Forests	2,955,866.26
5	Mixed Forests	4,811,134.80
6	Closed Shrublands	550,969.82
7	Open Shrublands	13,297,664.90
8	Woody Savannas	11,499, 310.33
9	Savannas	15,999,427.69
10	Grasslands	30,576,301.52
11	Permanent Wetlands	1,519,162.83
12	Croplands	11,805,688.37
	Total	107, 795,631.41 sq.km.

Section 3: Comparison of Satellite-derived SOS vs Fluxnet GPP-derived SOS

To evaluate how well satellite-derived phenology captures the ground-based start of season (SOS), we compared MODIS-derived SOS from the MCD12Q2.006 Land Cover Dynamics Yearly Global 500 m product with GPP-derived SOS from the Fluxnet2015. The SOS from MODIS was obtained from the *Greenup_1* band, which represents the date when EVI2 first crossed 15% of the segment EVI2 amplitude during the first growth cycle.

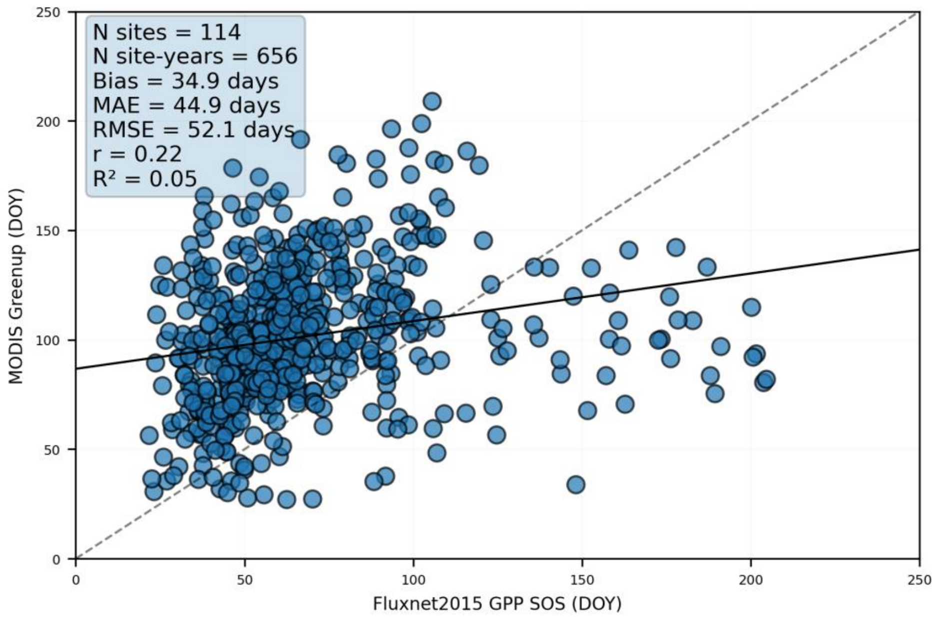

Figure S1. Scatter plot of MODIS SOS (Greenup_1 from MCD12Q2.006) versus Fluxnet2015 GPP-derived SOS for 114 sites and 656 site-years. The dashed line represents the 1:1 relationship. MODIS SOS systematically lags behind GPP-derived SOS by ~35 days, but relative interannual and spatial patterns are broadly preserved.

Across 114 sites and 656 site-years, MODIS SOS systematically lagged behind the GPP-based SOS, with an average bias of 34.9 days. These results indicate that the satellite-derived SOS captures mid-greenup rather than the true onset of photosynthesis. Despite this systematic offset, the MODIS metric preserves relative temporal patterns across sites and years. For this reason, while this comparison provides context on the potential differences between satellite-derived and GPP-derived SOS, the main analysis in this study relies on Fluxnet2015 GPP-derived SOS to quantify the influence of phenology on carbon flux. The detailed statistical metrics, including a scatter plot of MODIS versus GPP SOS is presented in Figure 1.

Section 4: Overview of phenology representation in major DGVMs

This table summarizes phenological representations on DGVMS as described in the literature and corresponding model documentation.

Table S3. Phenology representation in major DGVMs

DGVM	Primary controls on SOS/EOS	Key references
**CLM (Community Land Model)**	Air temperature, accumulated growing degree days (GDD), chilling requirements, soil moisture	(Lawrence et al., 2019)
**ORCHIDEE**	Temperature thresholds, photoperiod, soil moisture	(Krinner et al., 2005; Vuichard et al., 2019)
**JULES**	Temperature accumulation (GDD), soil moisture, calendar-based rules	(Best et al., 2011; Clark et al., 2011)
**LPJ-GUESS**	Temperature, soil moisture, carbon balance (leaf onset when C gain > cost)	(Sitch et al., 2003; Smith et al., 2001)

References

Best, M. J., Pryor, M., Clark, D. B., Rooney, G. G., Essery, R. . L. H., Ménard, C. B., Edwards, J. M., Hendry, M. A., Porson, A., Gedney, N., Mercado, L. M., Sitch, S., Blyth, E., Boucher, O., Cox, P. M., Grimmond, C. S. B., & Harding, R. J. (2011). The Joint UK Land Environment Simulator (JULES), model description – Part 1: Energy and water fluxes. *Geoscientific Model Development*, *4*(3), 677–699. https://doi.org/10.5194/GMD-4-677-2011

Clark, D. B., Mercado, L. M., Sitch, S., Jones, C. D., Gedney, N., Best, M. J., Pryor, M., Rooney, G. G., Essery, R. L. H., Blyth, E., Boucher, O., Harding, R. J., Huntingford, C., & Cox, P. M. (2011). The Joint UK Land Environment Simulator (JULES), model description – Part 2: Carbon fluxes and vegetation dynamics. *Geoscientific Model Development*, *4*(3), 701–722. https://doi.org/10.5194/GMD-4-701-2011

Krinner, G., Viovy, N., de Noblet-Ducoudré, N., Ogée, J., Polcher, J., Friedlingstein, P., Ciais, P., Sitch, S., & Prentice, I. C. (2005). A dynamic global vegetation model for studies of the coupled atmosphere-biosphere system. *Global Biogeochemical Cycles*, *19 (GB1015)*(1), 1–44. https://doi.org/10.1029/2003GB002199

Lawrence, D. M., Fisher, R. A., Koven, C. D., Oleson, K. W., Swenson, S. C., Bonan, G., Collier, N., Ghimire, B., van Kampenhout, L., Kennedy, D., Kluzek, E., Lawrence, P. J., Li, F., Li, H., Lombardozzi, D., Riley, W. J., Sacks, W. J., Shi, M., Vertenstein, M., … Zeng, X. (2019). The Community Land Model Version 5: Description of New Features, Benchmarking, and Impact of Forcing Uncertainty. *Journal of Advances in Modeling Earth Systems*, *11*(12), 4245–4287. https://doi.org/10.1029/2018MS001583;JOURNAL:JOURNAL:19422466;ISSUE:ISSUE:DOI

Sitch, S., Smith, B., Prentice, I. C., Arneth, A., Bondeau, A., Cramer, W., Kaplan, J. O., Levis, S., Lucht, W., Sykes, M. T., Thonicke, K., & Venevsky, S. (2003). Evaluation of ecosystem dynamics, plant geography and terrestrial carbon cycling in the LPJ dynamic global vegetation model. *Global Change Biology*, *9*(2), 161–185. https://doi.org/10.1046/J.1365-2486.2003.00569.X;JOURNAL:JOURNAL:13652486;WGROUP:STRING:PUBLICATION

Smith, B., Prentice, I. C., & Sykes, M. T. (2001). Representation of vegetation dynamics in the modelling of terrestrial ecosystems: comparing two contrasting approaches within European climate space. *Global Ecology and Biogeography*, *10*(6), 621–637. https://doi.org/10.1046/J.1466-822X.2001.T01-1-00256.X

Vuichard, N., Messina, P., Luyssaert, S., Guenet, B., Zaehle, S., Ghattas, J., Bastrikov, V., & Peylin, P. (2019). Accounting for carbon and nitrogen interactions in the global terrestrial ecosystem model ORCHIDEE (trunk version, rev 4999): multi-scale evaluation of gross primary production. *Geoscientific Model Development*, *12*(11), 4751–4779. https://doi.org/10.5194/GMD-12-4751-2019
